# FoxM1 promotes breast tumorigenesis by activating PDGF-A and forming a positive feedback loop with the PDGF/AKT signaling pathway

**DOI:** 10.18632/oncotarget.3596

**Published:** 2015-03-14

**Authors:** Guanzhen Yu, Aidong Zhou, Jianfei Xue, Chen Huang, Xia Zhang, Shin-Hyuk Kang, Wen-Tai Chiu, Christina Tan, Keping Xie, Jiejun Wang, Suyun Huang

**Affiliations:** ^1^ Departments of Neurosurgery, The University of Texas MD Anderson Cancer Center, Houston, TX, USA; ^2^ Departments of Gastroenterology, Hepatology, and Nutrition, The University of Texas MD Anderson Cancer Center, Houston, TX, USA; ^3^ Department of Medical Oncology, Changzheng Hospital, Shanghai, People's Republic of China; ^4^ Program in Cancer Biology, The University of Texas Graduate School of Biomedical Sciences at Houston, Houston, TX, USA

**Keywords:** breast cancer, FoxM1, PDGF-A, AKT, tumorigenesis

## Abstract

The autocrine platelet-derived growth factor (PDGF)/PDGF receptor (PDGFR) signaling pathway promotes breast cancer tumorigenesis, but the mechanisms for its dysregulation in breast cancer are largely unknown. In the study, we identified PDGF-A as a novel transcriptional target of FoxM1. FoxM1 directly binds to two sites in the promoter of *PDGF-A* and activates its transcription. Mutation of these FoxM1-binding sites diminished *PDGF-A* promoter activity. Increased FoxM1 resulted in the upregulation of PDGF-A, which led to activation of the AKT pathway and increased breast cancer cell proliferation and tumorigenesis, whereas knockdown of FoxM1 does the opposite. Blocking AKT activation with a phosphoinositide 3-kinase/AKT inhibitor decreased FoxM1-induced cell proliferation. Moreover, PDGF/AKT pathway upregulates the expression of FoxM1 in breast cancer cells. Knockdown of PDGF-A or blockade of AKT activation inhibited the expression of FoxM1 in breast cancer cells. Furthermore, expression of FoxM1 significantly correlated with the expression of PDGF-A and the activated AKT signaling pathway in human breast cancer specimens. Our study demonstrates a novel positive regulatory feedback loop between FoxM1 and the PDGF/AKT signaling pathway; this loop contributes to breast cancer cell growth and tumorigenesis.

## INTRODUCTION

Breast cancer is the most common malignancy and the leading cause of cancer-related death in women worldwide [1]. Breast cancer death rates have been declining in developed countries owing to early detection and improved treatment, but incidence and mortality rates have risen in developing areas [1], and thus, breast cancer still remains a substantial clinical challenge. Understanding the molecular mechanisms of the development and progression of breast cancer will highlight strategies for accurate diagnosis, early intervention, and effective therapies.

Several pathways have been implicated in breast cancer, including p53, epidermal growth factor receptor (EGFR), PDGF/PDGFR, PI3K/AKT, and mammalian target of rapamycin (mTOR) [2]. The AKT pathway is strongly activated during breast cancer development and progression. In one study, activation of AKT was observed in approximately 80% of breast cancer patients and was associated with poor disease-free survival [3]. Studies have shown that phosphorylation and activation of AKT kinase is often achieved by stimulation of the PDGF pathway through the binding of PDGFs to PDGFRs [4]. The activated PDGF pathway has been shown to prevent cancer cells from undergoing apoptosis during epithelial-mesenchymal transition and thus to promote breast cancer progression and metastasis [5, 6].

PDGFs are key growth factors in many kinds of tumors that are produced via autocrine or paracrine regulation and contribute to malignant progression [7]. One study found that the metastatic potential of mammary epithelial cancer cells requires an autocrine PDGF/PDGFR loop, and targeting the PDGF/PDGFR signaling was proved to therapeutically interfere with metastasis of breast cancers [6]. PDGF consists of several isoforms: alpha (A), beta (B), C, and D. Among these isoforms, PDGF-A has been shown to be over-expressed frequently in human breast tumors [8], and is an adverse prognostic factor in patients with advanced breast cancer [9]. However, little is known about the mechanism of PDGF-A dysregulation in human breast cancer.

FoxM1, a member of the Forkhead box transcription factor family, was first discovered as an oncogene, plays a key role in cell-cycle progression [10-12]. Aberrant expression of FoxM1 has been observed in the majority of human solid human tumors, including breast cancer [13-20]. Like the PDGF/AKT signaling pathway, which is also aberrantly activated in breast cancer, FoxM1 has been implicated in breast tumorigenesis. Moreover, *in vivo* and *in vitro* studies have also showed that FoxM1 depletion results in a significant reduction of cancer cell proliferation and tumor formation, suggesting that the consecutive expression of FoxM1 may act as a crucial factor for tumor growth [13, 21-23]. Aberrant activation or expression of FoxM1 also promotes the development of acquired drug resistance in various tumors, including breast cancer [24-28]. FoxM1 expression levels were elevated in 87% of breast cancer patients and the elevated FoxM1 levels were correlated with breast cancer development [29]. Enhanced expression of FoxM1 in breast cancer cells increased invasion and metastasis of these cells [30] and increased acquired drug resistance as well [26]. Moreover, several therapeutic targets for breast cancer, such as human epidermal growth factor receptor 2 and estrogen receptor alpha, may regulate the expression of FoxM1 [31, 32], which underlines the importance of FoxM1 as a therapeutic target in breast cancer treatment. However, the mechanisms for the roles of FoxM1 in breast cancer are largely unknown.

Since FoxM1 and the PDGF/AKT signaling pathway are both dysregulated in breast cancer and since both play important roles in breast tumorigenesis, in this study we explored the relationship between them to elucidate the responsible molecular mechanisms [6, 7, 30]. To our knowledge, we are the first to discover that FoxM1 activates the PDGF/AKT signaling pathway by directly transactivating PDGF-A. We also found that the PDGF/AKT signaling pathway increases FoxM1 expression. Therefore, our findings elucidate a novel FoxM1/PDGF/AKT regulatory feedback loop that promotes breast cancer cell growth and tumorigenesis.

## RESULTS

### FoxM1 activates the PDGF/AKT signaling pathway and promotes breast cancer cell growth

**Figure 1:**
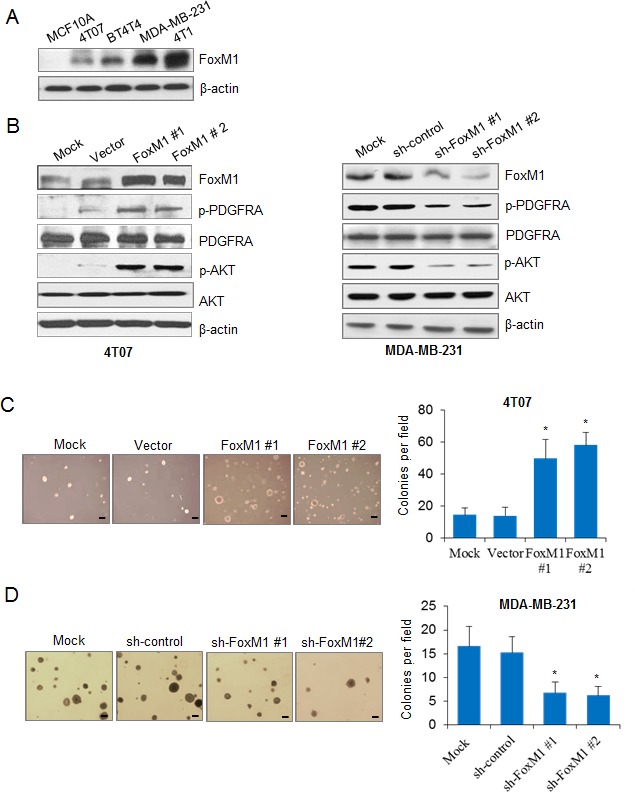
FoxM1 activates the PDGF/AKT signaling pathway and promotes breast cancer cell growth (A) Western blotting results show the expression of FoxM1 in different breast cancer cell lines. (B) Left panel, 4T07-FoxM1 stable cells were generated, and the expression of FoxM1, PDGFR-A, phospho-PDGFR-A (p-PDGFR-A), AKT, and phospho-AKT (p-AKT) were detected in the stable cells compared with the control cells. Right panel, MDA-MB-231-sh-FoxM1 stable cells were generated, and the expression of FoxM1, PDGFR-A, p-PDGFR-A, AKT, and p-AKT were detected by Western blotting. The MDA-MB-231-shControl stable cell line was used as a control. (C) 4T07 cells with or without enforced expression of FoxM1 were seeded in 6-well plates in triplicate at a density of 500 cells/well for 2 weeks. Representative images of cell growth are shown (left panel). Colonies with more than 50 cells were counted from six separate microscopic fields (Scale bar, 100 μm), and the average number of colony per field is shown (right panel). (D) MDA-MB-231-sh-FoxM1 stable cells were seeded in 6-well plates in triplicate at a density of 500 cells/well for 2 weeks. Representative images show the MDA-MB-231 cell growth (left panel). Colonies with more than 50 cells were counted from six separate microscopic fields, and the average number of colonies per field is shown (right panel). All tests were performed three times. *P < 0.05 compared with control groups.

We examined the expression levels of FoxM1 in two mouse mammary carcinoma cell lines (4T07 and 4T1), in two human mammary carcinoma cell lines (BT474 and MDA-MB-231), and in an immortalized normal human breast epithelial cell line (MCF 10A). FoxM1 levels were high in MDA-MB-231 and 4T1 cells, were relatively low in BT474 and 4T07 cells, and were barely detectable in MCF 10A cells (Fig. 1A).

We next explored the regulatory relationship between FoxM1 and the PDGF/AKT signaling pathway. To determine the effect of FoxM1 on the PDGF/AKT pathway, we used 4T07 cells with relatively low endogenous FoxM1 to generate 4T07-FoxM1 stable cell lines. We detected obvious elevation of PDGFR-A phosphorylation (Tyr754) in two different 4T07-FoxM1 stable cell lines but no alteration in the total PDGFR-A, suggesting that FoxM1 activated PDGFR-A (Fig. 1B, left panel). Furthermore, FoxM1 elevated the levels of phosphorylated AKT (Ser473), a downstream effector of the PDGF pathway, but the total protein levels remained the same (Fig. 1B, left panel). To determine the endogenous effect of FoxM1 on the PDGF pathway, we generated two MDA-MB-231-shFoxM1 stable cell lines using MDA-MB-231 cells and found that knockdown of FoxM1 decreased the activation and phosphorylation of PDGFR-A and AKT (Fig. 1B, right panel). These results indicate that FoxM1 activated the PDGF signaling pathway.

We next analyzed the roles of FoxM1 in breast tumor cell growth *in vitro*. Overexpression of FoxM1 in 4T07-FoxM1 stable cells significantly increased colony formation (Fig. 1C) and cell proliferation (Supplementary Fig. S1A), compared with control cells. Conversely, knocking down FoxM1 in MDA-MB-231-shFoxM1 cells significantly decreased colony formation (Fig. 1D) and cell proliferation (Supplementary Fig. S1B), compared with control cells. Together, these results suggest that FoxM1 activated the PDGF/AKT signaling pathway and promoted breast cancer cell growth.

### FoxM1 positively regulates PDGF-A expression in breast cancer cells

**Figure 2:**
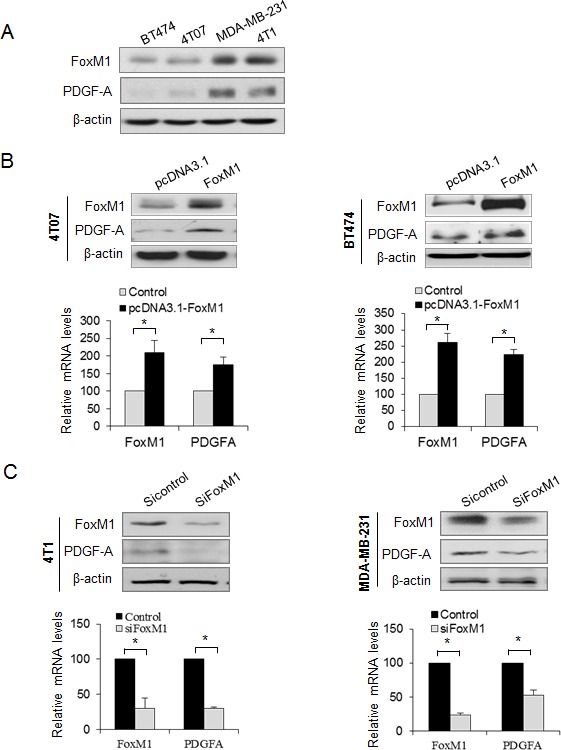
FoxM1 promotes PDGF-A expression in breast cancer cells (A) Correlation of FoxM1 and PDGF-A expression in different breast cancer cells revealed by Western blot analysis. (B) 4T07 cells and BT474 cells were transfected with pcDNA3.1-FoxM1 or pcDNA3.1 vector for 48 hours. Cell lysates were collected for Western blot analysis of FoxM1 and PDGF-A (upper panel). Relative mRNA levels of FoxM1 and PDGF-A were detected by quantitative real-time polymerase chain reaction (qRT-PCR) (lower panel). (C) 4T1 cells and MDA-MB-231 cells were transfected with SiFoxM1 or Sicontrol (50 nM) for 48 hours, and FoxM1 and PDGF-A protein or mRNA levels were detected by Western blot analysis (upper panel) and qRT-PCR (lower panel), respectively. The qRT-PCR results are expressed as percentage of FoxM1 or PDGF-A expression in the experiment groups versus the control groups. All experiments were performed three times. *P < 0.05.

The above results show that FoxM1 promoted PDGFR-A phosphorylation without altering the total PDGFR-A, suggesting that FoxM1 may activate the PDGF pathway through signals upstream of PDGFR-A. PDGF-A, as a homodimer or heterodimer with PDGF-B, can bind to PDGFR-A and promote PDGFR-A dimerization and tyrosine phosphorylation in the cytoplasm, which then activates several signaling cascades. We therefore analyzed the correlation of FoxM1 with PDGF-A in different breast cancer cell lines and found that PDGF-A expression level was positively correlated with FoxM1 in these cell lines (Fig. 2A). We next overexpressed FoxM1 in two cell lines, BT474 and 4T07, which both have low levels of endogenous FoxM1, and found that PDGF-A was dramatically elevated in both cell lines (Fig. 2B, upper panel). Also, 4T07-FoxM1 stable cells had higher levels of PDGF-A expression and secretion than did the control cells (Supplementary Fig. S2A). FoxM1 elevates PDGF-A levels via transcription activation because the mRNA level of PDGF-A was increased in both 4T07 and BT474 cells (Fig. 2B, lower panels). Conversely, knocking down FoxM1 in 4T1 and MDA-MB-231 cells, which have high levels of endogenous FoxM1, significantly downregulated both PDGF-A protein and mRNA expression (Fig. 2C). Furthermore, MDA-MB-231-shFoxM1 stable cells had lower levels of PDGF-A expression and secretion than did the control stable cells (Supplementary Fig. S2B). These results indicate that FoxM1 regulated PDGF-A in breast cancer cells.

### *PDGF-A* is a direct transcriptional target of FoxM1

**Figure 3:**
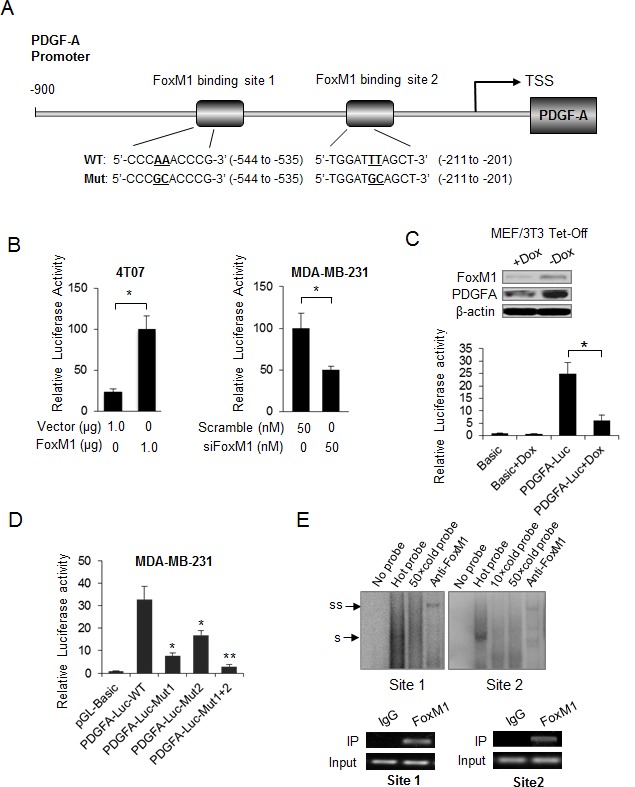
PDGF-A is a direct transcriptional target of FoxM1 (A) Diagram shows the sequence and position of two putative FoxM1-binding elements in the *PDGF-A* promoter. Abbreviations: TSS, transcriptional start site; WT, wild type; Mut, mutant type. (B) Left panel, 4T07 cells were cotransfected with the *PDGF-A* promoter reporter, pRL-TK, and pcDNA3.1-FoxM1 or pcDNA 3.1; right panel, MDA-MB-231 cells were cotransfected with the *PDGF-A* promoter reporter, pRL-TK, and SiFoxM1 or Sicontrol (50 nM). Forty-eight hours after transfection, the cells were collected, and the relative *PDGF-A* promoter activities were measured. Data are from three independent assays. *P < 0.05. (C) Upper panel, the MEF/3T3 Tet-Off cells expressing FoxM1 were treated with doxycycline and hydrochloride (Dox), and the protein levels of FoxM1 and PDGF-A were detected by Western blot analysis; middle panel, MEF/3T3 Tet-Off cells expressing FoxM1 were transfected with the *PDGF-A* promoter reporter or the control reporter and then treated with Dox for 8 hours. Cells were lysed 48 hours after transfection, and the relative *PDGF-A* promoter activities were measured. *P < 0.05. (D) Reporter plasmids harboring the wild-type *PDGF-A* promoter or the corresponding mutant promoter in the FoxM1-binding sites were transfected into MDA-MB-231 cells, and the relative promoter activities were measured as above. Data are from three independent assays. *P < 0.05; **P < 0.01. (E) Upper panel, electrophoresis mobility shift assay (EMSA) results show the *in vitro* binding of FoxM1 with *PDGF-A* promoter. Probes harboring the potential FoxM1-binding sites (site 1 or site 2) were labeled with γ-p ^32^ATP and then were incubated with the MDA-MB-231 nuclear extract. For the supershift assay, FoxM1 antibody (1 μg) was added to the reaction. s, shift; ss, super shift. Lower panel, the chromatin immunoprecipitation (ChIP) assay results show the *in vivo* binding of FoxM1 to the *PDGF-A* promoter. MDA-MB-231 cell lysis was immunoprecipitated using an anti-FoxM1 antibody or immunoglobulin G. The resulting samples were subjected to semiquantitative polymerase chain reaction using the site-specific primers.

To determine whether FoxM1 transcriptionally regulates *PDGF-A* directly, we searched the promoter region of human *PDGF-A* (NM_002607) for the consensus-binding site of FoxM1. Two potential FoxM1-binding sites were observed in a 900-bp region upstream of the transcriptional start site (Fig. 3A). We then assessed the effect of altering FoxM1 on the activity of the *PDGF-A* promoter in breast cancer cells. We found that the activity of the *PDGF-A* promoter was increased by FoxM1 overexpression in 4T07 cells and was decreased by FoxM1 knockdown in MDA-MB-231 cells (Fig. 3B, Supplementary Fig. S3).

We next generated a Tet-Off–based FoxM1 inducible MEF/3T3 cell line to control FoxM1 expression. FoxM1 was repressed by culturing FoxM1-transfected MEF/3T3 Tet-Off cells in the presence of doxycycline hydrochloride, and PDGF-A was also dramatically decreased after FoxM1 suppression (Fig. 3C, upper panel). We found that doxycycline hydrochloride suppressed PDGF-A via transcription inhibition because doxycycline hydrochloride dramatically decreased the promoter activity of *PDGF-A* (Fig. 3C, lower panel).

To confirm the roles of the two putative predictiveFoxM1-binding sites in mediating the response of the *PDGF-A* promoter to FoxM1, we constructed two reporter plasmids with a single mutation in site 1 or site 2 and another reporter plasmid with mutations in both sites (Fig. 3A). The results show that mutation of site 1 or site 2 significantly decreased *PDGF-A* promoter activity, and simultaneous mutation of both sites almost abolished the promoter activity (Fig. 3D).

We further investigated whether FoxM1 activates *PDGF-A* transcription by directly binding to these sites. We performed EMSA assays by incubating the labeled probes harboring site 1 or site 2 with the nuclear extracts from MDA-MB-231 cells. A shift band was detected using either of the two labeled probes, and the shift band disappeared in samples with 50 times molar excess of the unlabeled probe (Fig. 3E, upper panel). Moreover, adding FoxM1 antibody further slowed the migration of the shift band (Fig. 3E, upper panel), suggesting that FoxM1 binds to site 1 and site 2 in the *PDGF-A* promoter. To determine *in vivo* the binding site of FoxM1 to the *PDGF-A* promoter, we performed a ChIP assay in MDA-MB-231 cells, which showed that FoxM1 bound to both site 1 and site 2 in the *PDGF-A* promoter (Fig. 3E, lower panel). Taken together, the above results indicate that FoxM1 bound directly to the *PDGF-A* promoter and activated *PDGF-A* transcription in breast cancer cells.

### FoxM1 activates the AKT pathway and promotes breast cancer cell growth through PDGF-A

**Figure 4:**
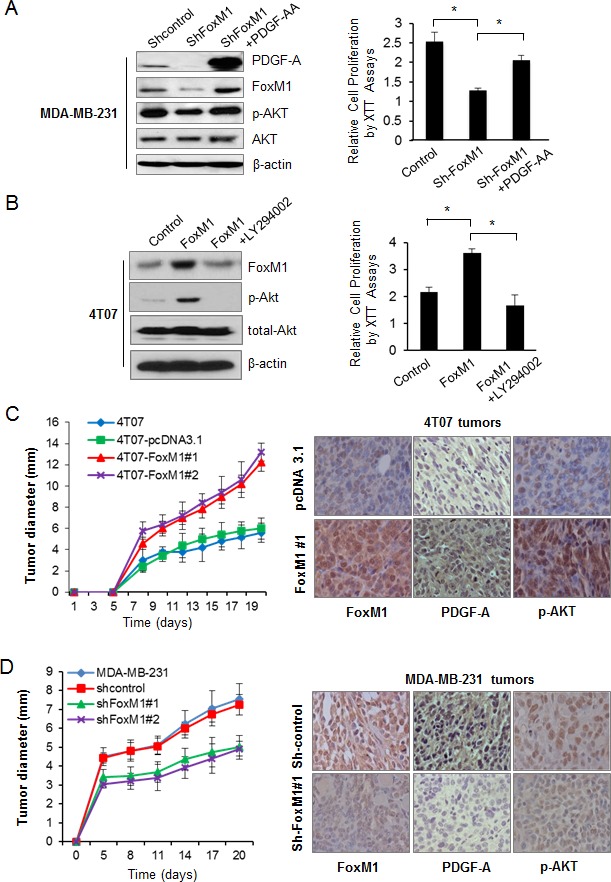
Forkhead box M1 (FoxM1)/PDGF/AKT pathway promotes tumorigenesis of breast cancer cells (A) Left panel, MDA-MB-231-shFoxM1 stable cells were treated with PDGF-AA for 24 hours, and the protein levels of FoxM1, PDGF-A, p-AKT, and total AKT were analyzed by Western blot analysis. Right panel, cell proliferation of MDA-MB-231-shFoxM1 treated with or without PDGF-AA was analyzed by XTT assay. (B) Left panel, 4T07-FoxM1 stable cells were treated with LY294002 for 4 hours, and cell lysis was subjected to Western blot analysis using the indicated antibodies. Right panel, cell proliferation of 4T07-FoxM1 stable cells treated with or without LY294002 was analyzed by XTT assay. Data are from three independent experiments. *P < 0.05. (C) 4T07 stable cells with or without FoxM1 overexpression (1×10^5^ per mouse) were injected into the mammary fat pad of nude mice (n = 5 for each group), and the tumor diameter (mm) was examined at different time points (left panel). The formed tumors were sectioned, and expression of FoxM1, PDGF-A, and phospho-AKT (p-AKT) was detected by immunohistochemical analysis (right panel). (D) MDA-MB-231 stable cells (5×10^6^ per mouse) with or without shRNA-mediated depletion of FoxM1 were injected into the mammary fat pad of nude mice (n = 10 for each group). The tumor diameter was examined at different time points (left panel). The expression of FoxM1, PDGF-A, and p-AKT was analyzed by immunohistochemical analysis (right panel).

Our data indicate that FoxM1 upregulated the levels of PDGF-A and phospho-AKT in breast cancer cells (Fig. 1B and D, Supplementary Fig. S4); therefore, we investigated whether the activation of the AKT pathway by FoxM1 depends on PDGF-A. In BT474 cells, FoxM1 transfection significantly upregulated PDGF-A expression; however, this effect was reversed by PDGF-A shRNA transfection (Supplementary Fig. S5, left panel). Conversely, in MDA-MB-231 cells, knockdown of FoxM1 by shRNA decreased the phospho-AKT level, and exogenous PDGF-AA reversed the effect of FoxM1 downregulation on the activation of the AKT signaling pathway (Fig. 4A, left panel).

We next determined the role of FoxM1 in the proliferation of breast cancer cells through the PDGF-A/AKT pathway. BT474 cell growth significantly increased after overexpression of FoxM1 but was suppressed after simultaneous knockdown of PDGF-A (Supplementary Fig. S5, right panel). Conversely, MDA-MB-231 cell growth was inhibited after FoxM1 knockdown, but this inhibition was largely reversed by exogenous PDGF-AA treatment (Fig. 4A, right panel). Furthermore, the role of FoxM1 in cell proliferation also depends on the PI3K/AKT pathway because treating 4T07-FoxM1 cells with the PI3K/AKT pathway inhibitor LY294002 reduced the level of phospho-AKT (Fig. 4B, left panel) and suppressed the growth of the cells (Fig. 4B, right panel), which indicates that the role of FoxM1 in cell proliferation also depends on the PI3K/AKT pathway. Taken together, these results indicate that FoxM1 activated the AKT signaling pathway and promoted breast cancer cell growth through PDGF-A.

### FoxM1/PDGF/AKT pathway promotes tumorigenesis of breast cancer cells

To determine the roles of FoxM1 in the tumorigenesis of breast cancer cells by activating the PDGF/AKT pathway, we injected 4T07-FoxM1 cells into the mammary fat pads of nude mice. FoxM1 overexpression promoted tumorigenesis of the 4T07 cells: the tumors of mice injected with 4T07-FoxM1 cells were significantly larger than those of mice injected with the control 4T07 cells (Fig. 4C, left panel). To determine the *in vivo* function of FoxM1 in tumorigenesis of breast cancer cells, we performed FoxM1 loss-of-function studies by injecting 4T1-shFoxM1 and MDA-MB-231-shFoxM1 cells into the mammary fat pads of nude mice. We found that knockdown of FoxM1 inhibited tumorigenesis of 4T1 cells; the tumors of mice injected with 4T1-shFoxM1 cells were significantly smaller than those of mice injected with 4T1 or 4T1-sh-control cells (Supplementary Fig. S6, left panel). Likewise, knockdown of FoxM1 also significantly inhibited tumorigenesis in human breast cancer MDA-MB-231 cells (Fig. 4D, left panel).

We next detected the expression of FoxM1, PDGF-A, and phospho-AKT (Ser473) using immunohistochemical assay in the tumors from the above *in vivo* experiments. Tumors from 4T07-FoxM1 cells had higher levels of FoxM1, PDGF-A, and phospho-AKT than did tumors from 4T07-pcDNA-control cells (Fig. 4C, right panel). Furthermore, we also examined the expression of Ki-67, a biomarker of tumor cell proliferation; Ki-67 staining was significantly increasedin the tumors from 4T07-FoxM1 cells that that from the control cells (Supplementary Fig. S7A). In contrast, tumors from 4T1-shFoxM1 and MDA-MB-231-shFoxM1 cells had lower levels of FoxM1, PDGF-A, and phospho-AKT than did tumors from the sh-control cells (Fig. 4D and Supplementary Fig. S6, right panel). Likewise, the Ki-67 staining was also decreased in the tumors after FoxM1 knockdown compared with the controls (Supplementary Fig. S7B). Taken together, our results suggest that FoxM1 promoted breast tumorigenesis, at least partly, by elevating PDGF-A expression and activating the AKT pathway.

### PDGF-A regulates FoxM1 through the AKT pathway

**Figure 5:**
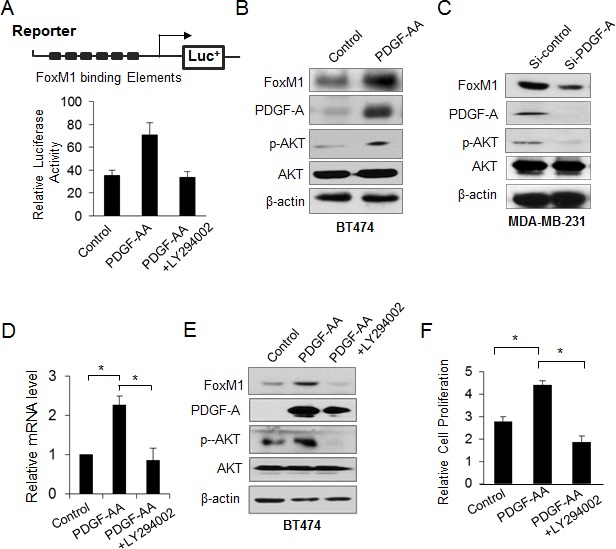
FoxM1 is activated by PDGF/AKT signaling pathway (A) BT474 cells were cotransfected with reporter construct harboring the consensus FoxM1 responsive element and pRL-TK and then were treated with PDGF-AA (50 μg/L) or PDGF-AA plus LY294002 (25 μM) for 24 hours. Forty-eight hours later, cells were lysed, and the relative luciferase activity was measured. Data are from three independent assays. *P ≤ 0.05. (B) BT474 cells were treated with PDGF-AA for 24 hours, and the expression of FoxM1, PDGF-A, phospho-AKT (p-AKT), and total AKT was analyzed by Western blotting. (C) MDA-MB-231 cells were transfected with PDGF-A siRNA or control siRNA (50 nM), and the expression of FoxM1, PDGF-A, p-AKT, and total AKT was analyzed by Western blotting. (D and E) BT474 cells were treated with PDGF-AA (50 μg/L) or PDGF-AA plus LY294002 (25 μM) for 24 hours. The expression of FoxM1 was detected by real-time PCR (D) and western blotting (E). (F) BT474 cell proliferation was analyzed by XTT assay after cells were treated with PDGF-AA or PDGF-AA plus LY294002. Data are from three independent assays. *P <0.05.

To further reveal the crosstalk between FoxM1 and the PDGF pathway, we examined the effect of the PDGF/AKT signaling pathway on FoxM1. Using a reporter plasmid harboring the consensus FoxM1-binding elements, we found that PDGF-A activated FoxM1-mediated transcription and that this activation was blocked by LY294002, an inhibitor of the AKT pathway (Fig. 5A). To determine the effect of PDGF-A on FoxM1 expression level, we treated BT474 cells with PDGF-AA and found elevated FoxM1 levels (Fig. 5B). In contrast, knocking down PDGF-A in MDA-MB-231 cells decreased the expression of FoxM1 in the cells (Fig. 5C). Moreover, the levels of phospho-AKT were increased after PDGF-AA treatment but were decreased by knockdown of PDGF-A in BT474 and MDA-MB-231 breast cancer cells (Fig. 5B and C). Furthermore, LY294002 blocked the ability of PDGF-AA to induce FoxM1 expression in BT474 cells (Fig. 5D and E). Also, LY294002 blocked the cell growth induced by PDGF-AA (Fig. 5D, right panel). Together, these results indicate that PDGF-A regulated FoxM1 through the AKT pathway.

### FoxM1 expression positively correlated with the expression of PDGF-A and phospho-AKT in human breast tumors

**Figure 6:**
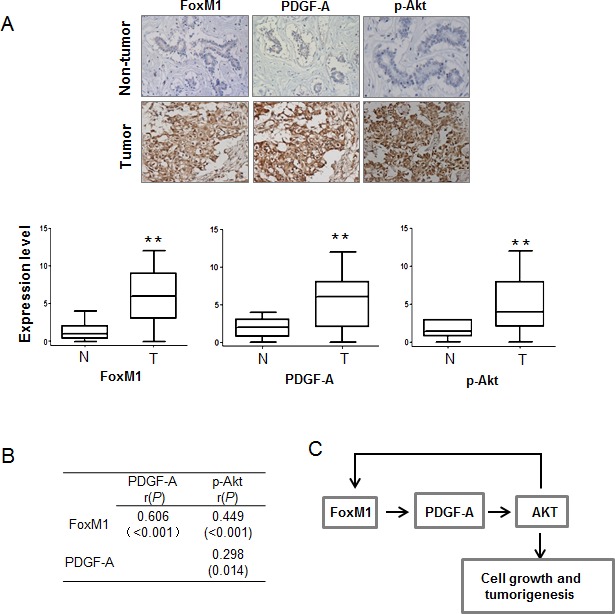
FoxM1, PDGF-A, and phospho-AKT (p-AKT) are positively correlated in human breast cancer samples (A) Upper panel, expression of FoxM1, PDGF-A, and p-AKT was examined by immunohistochemical assay in 67 breast cancer specimens and 10 adjacent normal tissues. Images shown are representatives of the examined samples. Magnification 200×. Lower panel, the level of FoxM1, PDGF-A, and p-AKT staining was scored from 0 to 12. The expression of FoxM1, PDGF-A, and p-AKT between non-tumor (N) and tumor specimens (T) were evaluated. **P < 0.01. (B) The expression correlations among FoxM1, PDGF-A, and p-AKT were significant as determined by Pearson's correlation test. *r*: Pearson correlation coefficient. (C) Schematic diagram shows the mechanism underlying FoxM1, PDGF-A, and AKT: FoxM1 transcriptionally activates PDGF-A and the AKT signaling pathway; in return, the activated AKT pathway upregulates FoxM1 expression, which forms a positive feedback loop and promotes breast cancer cell growth and tumorigenesis.

To determine whether our findings have clinical relevance in human breast cancer, we investigated the expression of FoxM1, PDGF-A, and phospho-AKT in human breast tumor samples. We analyzed the significance of the FoxM1/PDGF-A/AKT axis in a panel of 67 human breast cancer specimens and 10 adjacent non-tumor tissues. The expression levels of FoxM1, PDGF-A, and phospho-AKT were significantly higher in tumor tissues than in non-tumor tissues (Fig. 6A). The expression levels of FoxM1 directly correlated with those of PDGF-A and phospho-AKT (Fig. 6B). These results further support the essential role of FoxM1 in regulating PDGF-A induction and AKT activation in human breast cancers and the role of the FoxM1/PDGF-A/AKT axis in the pathogenesis of human breast cancer. Collectively, our findings demonstrate a positive regulatory feedback loop in which FoxM1 transactivates PDGF-A expression and the AKT signaling pathway, events that in turn elevate FoxM1 expression levels and promote breast tumor cell growth and tumorigenesis (Fig. 6C).

## DISCUSSION

Our study demonstrates strong evidence supporting a critical role for FoxM1 in regulating breast cell tumorigenicity. We showed that FoxM1 enhances the survival and growth of human breast cancer cells through the PDGF/AKT signaling pathway. Specifically, FoxM1 stimulates the transcription of PDGF-A by directly binding to its promoter at two sites, which in turn leads to the activation of the AKT pathway, one of the most important pathways underlying breast tumorigenesis. We also found that the PDGF/AKT pathway elevates FoxM1 expression and that the level of FoxM1 is significantly correlated with that of PDGF-A and with the activity of the AKT pathway in human breast cancers. The regulatory feedback between FoxM1 and the PDGF/AKT pathway revealed in our study may represent a critical mechanism for the proliferation and tumorigenesis of human breast cancer (Fig. 6D).

Studies have shown that the formation and progression of breast cancer depend on multiple interactions of several proteins and signaling pathways [6, 35]. Our study supports the notion that FoxM1 overexpression, upregulation of PDGF-A, and activation of the PI3K/AKT pathway favor breast cancer cell proliferation and tumorigenesis. Studies have shown that FoxM1 stimulates the transcription of estrogen receptor-alpha and is significantly correlated with estrogen receptor-alpha in breast cancer cells [17]. Our study demonstrated that FoxM1 activates the PDGF/AKT signaling pathway by stimulating the transcription of PDGF-A in breast cancer cells. Moreover, knockdown of PDGF-A blocks FoxM1-induced cell proliferation. Therefore, our findings elucidate a hitherto unexplored mechanism for FoxM1 in breast cancer progression.

Previous studies have shown that the autocrine PDGF/PDGFR signaling pathway promotes breast cancer progression [5] and that inhibiting PDGFR-A expression or activity by reducing its phosphorylation is an effective strategy for molecular therapy in breast cancer or other tumors [36]. PDGF-A, an important ligand of PDGFR, induces PDGFR phosphorylation and activation. Although previous studies have shown that PDGF-A is dysregulated in breast cancer and is correlated with cancer stage and rate of progression in breast cancer patients [9], the mechanisms for this dysregulation are largely unknown. Our results demonstrate that PDGF-A is a direct transcriptional target of FoxM1 and that the expression of FoxM1 is significantly correlated with that of PDGF-A in breast cancer. Moreover, FoxM1 activates the PDGF/PDGFR pathway in breast cancer cells by stimulating the transcription of PDGF-A. Thus, our findings reveal a novel mechanism for PDGF-A dysregulation in breast cancer and for the activation of PDGF/PDGFR signaling in breast tumor progression.

We discovered a novel positive regulatory feedback loop; these loops are a common mechanism for consecutive activation of factors or signaling pathways in tumor progression. We found that FoxM1 activates the AKT pathway by stimulating the PDGF/PDGFR pathway through PDGF-A. Moreover, we also found that the PDGF/AKT pathway activates FoxM1 expression in breast cancer cells. This regulatory feedback loop between FoxM1 and PDGF/AKT maintains the consecutive activation of the AKT signaling pathway, which may be a critical mechanism for breast tumor progression.

In summary, our findings show crosstalk between FoxM1 and the PDGF/AKT signaling pathway in human breast cancer. Owing to the great importance of the FoxM1/PDGF-A/AKT axis in human cancers, our findings strongly suggest that targeting FoxM1 may be an alternative therapeutic strategy for breast cancer.

## MATERIALS AND METHODS

### Cell culture and reagents

BT474, MDA-MB-231, 4T07, and 4T1 breast cancer cells were cultured in Dulbecco's modified Eagle's medium supplemented with 10% fetal bovine serum, 1% penicillin/streptomycin, and 1% glutaMAX (Invitrogen). MCF 10A cells were cultured in Dulbecco's modified Eagle's medium/F-12 supplemented with 5% heat-inactivated horse serum (Life Technologies), insulin (10 μg/mL, Life Technologies), cholera toxin (100 ng/mL, Sigma-Aldrich), hydrocortisone (0.5 μg/mL, Sigma-Aldrich), recombinant epidermal growth factor (20 ng/mL, Life Technologies), and 1% penicillin/streptomycin. The MEF/3T3 Tet-Off cell line from Clontech was used to stably express the tetracycline-controlled transactivator. Recombinant human PDGF-AA was purchased from Sigma-Aldrich, and the final treatment concentration was 10 ng/mL. Doxycycline hydrochloride was purchased from Sigma-Aldrich and was used at a final concentration of 1 μg/mL. The specific phosphoinositide 3-kinase (PI3K)/AKT inhibitor LY294002 was purchased from Cell Signaling Technology and was used at a final concentration of 10 μM.

### Human tissue specimens and patients

Human breast cancer tissue microarray (BR1503a) was purchased from US Biomax. Sixty-seven patients were enrolled in this study. None of these patients underwent preoperative chemotherapy or radiation therapy. The median age was 49 years (range, 19-69 years). Of the 67 patients, 7 (10.4%) presented with intraductal carcinoma (tumor-node-metastasis stage (TNM), TisN0M0), and 60 patients (89.6%) had invasive ductal carcinoma. Of these 60 patients, 7 (11.7%) had TNM stage I tumors, 34 had (56.7%) TNM stage II tumors, 14 (23.3%) had TNM stage III tumors, and 5 (8.3%) had TNM stage IV tumors.

### Plasmids, siRNA, and transfection

The pcDNA3.1-FoxM1 and pSilencer-shFoxM1 plasmids were constructed as described previously [16]. pSilencer-shFoxM1 and pSilencer-shPDGF-A were constructed using the following target sequences: FoxM1, 5′-CUCUUCUCCCUCAGAUAUA-3′; PDGF-A, 5′-CUGAAUCCGGAUUAUCGGGAA-3′. pSilencer-shcontrol expressing a hairpin shRNA with limited homology to any known sequences in the human, mouse, and rat genomes was also constructed and was used as a control. Mutagenesis in the *PDGF-A* promoter was introduced using the QuikChange site-directed mutagenesis kit (Agilent Technologies).

The siGENOME SMART pool siRNA targeting FoxM1 and the scramble siRNA were purchased from Dharmacon. For siRNA transfection, cells were transfected into FoxM1 siRNA or scramble siRNA at a final concentration of 50 nM using Lipofectamine RNAiMAX transfection reagent (Life Technologies), according to the manufacturer's instructions.

### Western blot analysis

Cells were lysed using radioimmunoprecipitation assay buffer (Tris-hydrochloric acid (50 mM), pH 8.0; sodium chloride (150 mM); 1% NP-40; 0.5% sodium deoxycholate; 0.1% sodium dodecyl sulfate; and ethylenediaminetetraacetic acid (2 mM)) containing protease inhibitor cocktail (Sigma-Aldrich). Protein extracts were separated by sodium dodecyl sulfate polyacrylamide gel electrophoresis (SDS-PAGE) and transferred to polyvinylidene difluoride membranes (EMD Millipore). The membranes were incubated with antibodies against FoxM1 (sc-500, Santa Cruz Biotechnology), PDGF-A (NBP1-19781, Novus Biologicals), PDGFR-A (sc-338, Santa Cruz Biotechnology), phospho-PDGFRA (Tyr 754) (sc-12911, Santa Cruz Biotechnology), AKT (#9272, Cell Signaling Technology), phospho-AKT (Ser473) (#9271, Cell Signaling Technology). β-actin was used as a loading control.

### Cell viability and colony formation assays

We performed cell viability assays using a TACS XTT assay kit (R&D Systems). Briefly, cells were plated in 96-well plates (1×10^4^/well) and were treated with PDGF-AA or LY294002 for 24 hours. We added 50 μL of XTT working solution (combining XTT reagent with XTT activator) to each well. After incubating the cells for 6 hours, we obtained the absorbance values at 490 nm with a reference correction at 630 nm in an enzyme-linked immunosorbent assay plate reader (Thermo Scientific). Data were obtained from three independent assays in triplicate.

We performed colony formation assays as described previously [16]. Only colonies containing more than 50 cells were scored. Data shown are mean number of colonies observed in six randomly chosen microscopic fields (magnification ×200).

### Promoter reporters and luciferase reporter assay

We used *PDGF-A* promoter reporter plasmids [33], mutant *PDGF-A* promoter reporter plasmids, or FoxM1-dependent luciferase promoter reporter plasmids [23]. Cells in 24-well plates were transfected with each reporter plasmid and *Renilla* luciferase (pRL-TK) vector plasmid. Forty-eight hours after transfection, cells were harvested for firefly/*Renilla* luciferase assays using the Dual-Glo luciferase reporter assay system (Promega). Luciferase activities were normalized to the cotransfected pRL-TK plasmid. All experiments were performed at least twice in triplicate.

### Electrophoresis mobility shift assay (EMSA)

We performed electrophoresis mobility shift assays as described previously [34]. For the supershift assay, we added antibody against human FoxM1 (2 μL; sc-500, Santa Cruz Biotechnology) to the protein-DNA complex. The protein-DNA complexes were separated in 4% native acrylamide gels and visualized by autoradiography. Probes used in the EMSA analysis are shown in Supplementary Table S1.

### Chromatin immunoprecipitation (ChIP) assay

We performed ChIP assays using a SimpleChIP Enzymatic Chromatin IP kit (Cell Signaling Technology), according to the manufacturer's instructions. Briefly, MDA-MB-231 cells in a 100-mm dish were cross-linked with 1% formaldehyde and then digested by micrococcal nuclease for 20 minutes at 37°C. The cross-linked protein-DNA complexes were immunoprecipitated using an antibody against FoxM1. DNA was purified and then subjected to semiquantitative PCR analysis using site-specific primers (Supplementary Table S1).

### Mammary fat pad tumor growth

Female athymic BALB/c nude mice were purchased from the National Cancer Institute and were maintained in institutional facilities approved by the Association for Assessment and Accreditation of Laboratory Animal Care. Breast cancer cells were injected into the mammary fat pads of nude mice, and the diameters of the resulting tumors were measured once every 5 days. Three to four weeks later, the mice were killed, and the tumors were dissected. Tumor samples were fixed in formalin for hematoxylin and eosin and immunohistochemical analysis.

### Statistical analysis

Statistical analysis was performed with SPSS version 16.0. We used a two-tailed Student *t* test to determine the significance of the *in vitro* results and a one-way analysis of variance to determine the significance of the *in vivo* data. We determined the significance of differences in the human tissue data using Pearson's correlation test. *P* values less than 0.05 were considered statistically significant.

## SUPPLEMENTARY MATERIALS, FIGURES, TABLES


