# Supplementary material for: FoxM1 promotes breast tumorigenesis by activating PDGF-A and forming a positive feedback loop with the PDGF/AKT signaling pathway
Source: Oncotarget. 2015 Mar 14;6(13):11281–94. doi: 10.18632/oncotarget.3596 (PMC4484456; doi:10.18632/oncotarget.3596)
Supplement: Supplementary file 1 [file oncotarget-06-11281-s001.pdf]

FoxM1 promotes breast tumorigenesis by activating PDGF-A and forming a positive feedback loop with the PDGF/AKT signaling pathway

Supplementary Material

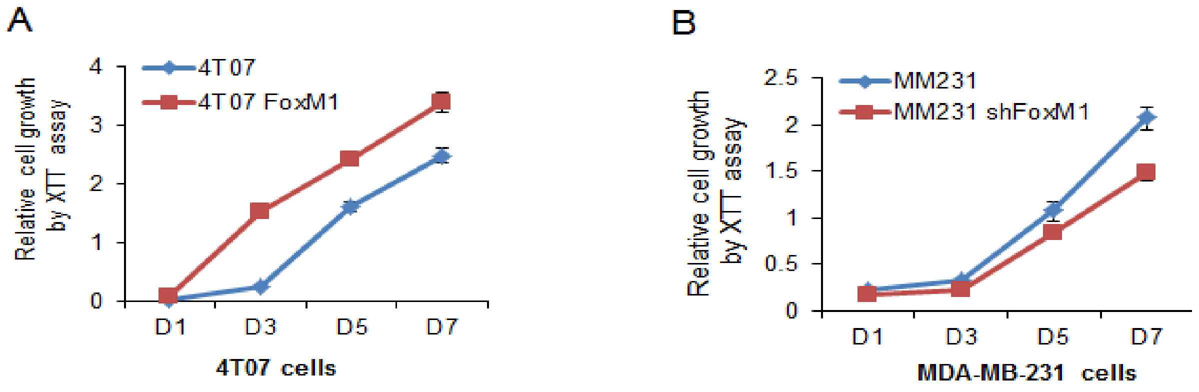

Supplementary Figure 1: XTT assays show the effect of FoxM1 on 4T07 and MDA-MB-231 cell proliferation. The 4T07-FoxM1 stable cells (A) or MDA-MB-231-shFoxM1 stable cells (B) were seeded in 6-well plates, and the relative cell proliferation was measured at the indicated days by XTT assay.

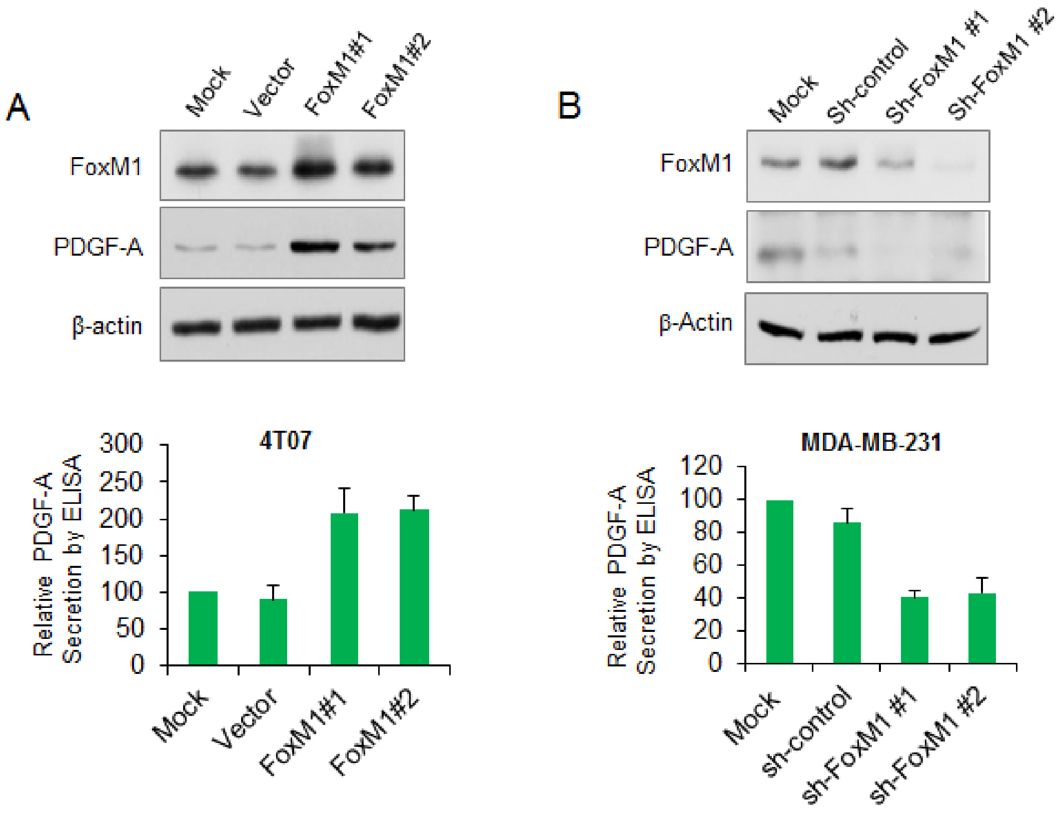

Supplementary Figure 2: Effect of FoxM1 on PDGF-A expression and secretion in breast cancer cells. A, Overexpression of FoxM1 on PDGF-A expression (up panel) and secretion (down panel) in 4T07 cells by western blot and Elisa assays, respectively. Two 4T07-FoxM1 stable colonies (#1 and #2) were analyzed. 4T07 cells with no transfection (Mock) or transfected with a pcDNA3.1 vector (Vector) were used as controls. Data were from two independent assays. B, Knockdown of FoxM1 on PDGFA expression (up panel) and secretion (down panel) in MDA-MB-231cells. Two different MDA-MB-231-shFoxM1 stable colonies (#1 and #2) were analyzed. Cells with no transfection (Mock) or transfected with a scramble shRNA (sh-control) were used as controls.

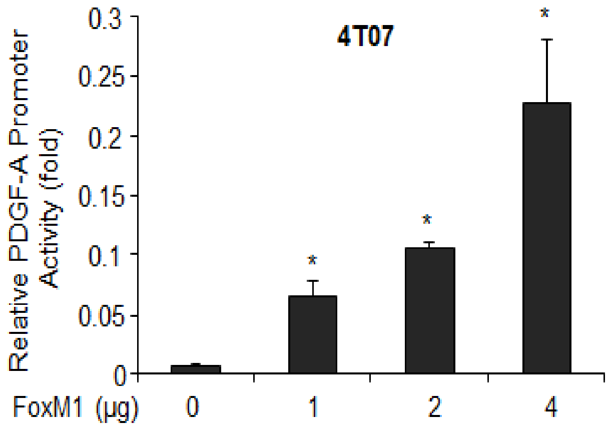

Supplementary Figure 3: Dual luciferase assay shows that FoxM1 activates PDGFA promoter in a dose dependent manner in 4T07 cells. 4T07 cells were transfected with different amount of FoxM1 expression plasmid and a reporter plasmid harboring the PDGF-A promoter. The relative promoter activities were measured 48 h after transfection. Data were from two independent assays. *p <0.05.

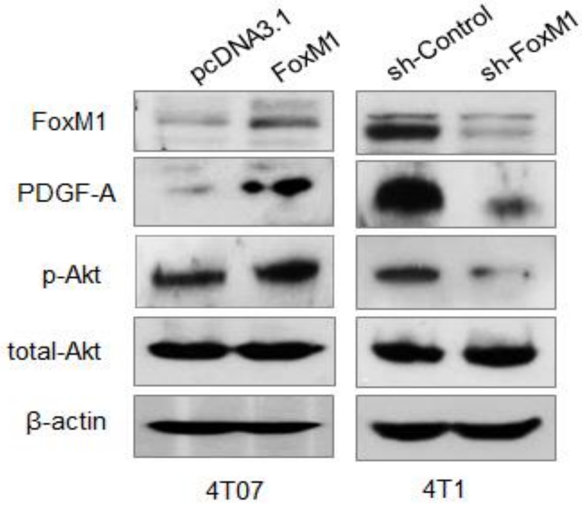

Supplementary Figure 4: FoxM1 activates the PDGF/AKT pathway. 4T07-FoxM1 and 4T1-sh-FoxM1 stable cells were Analyzed by western blot using the indicated antibodies. β-actin was used as an internal control.

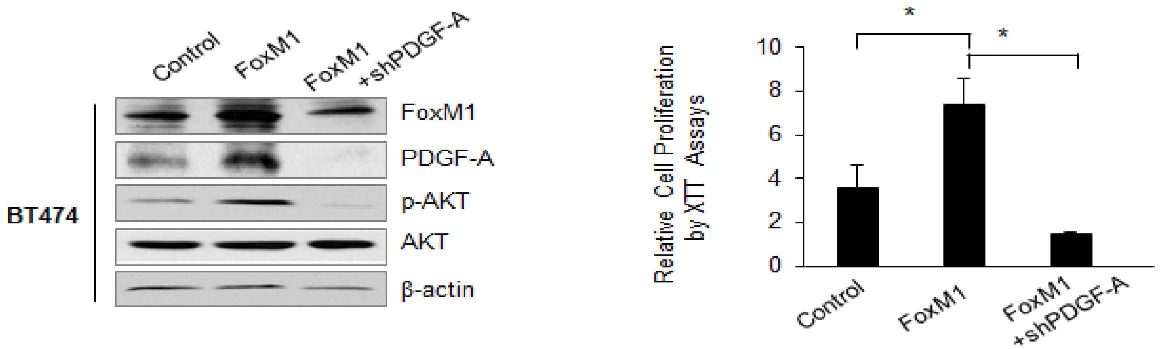

Supplementary Figure 5: Left panel, BT474-FoxM1 and BT474-FoxM1-shPDGF-A stable cell lines were generated, and the protein levels of FoxM1, PDGF-A, phospho-AKT (p-AKT), and total AKT were detected by Western blot analysis. Right panel, we used an XTT assay to detect cell proliferation of BT474-FoxM1 and BT474-FoxM1-shPDGF-A. Data are from three independent experiments. *$P < 0.05$.

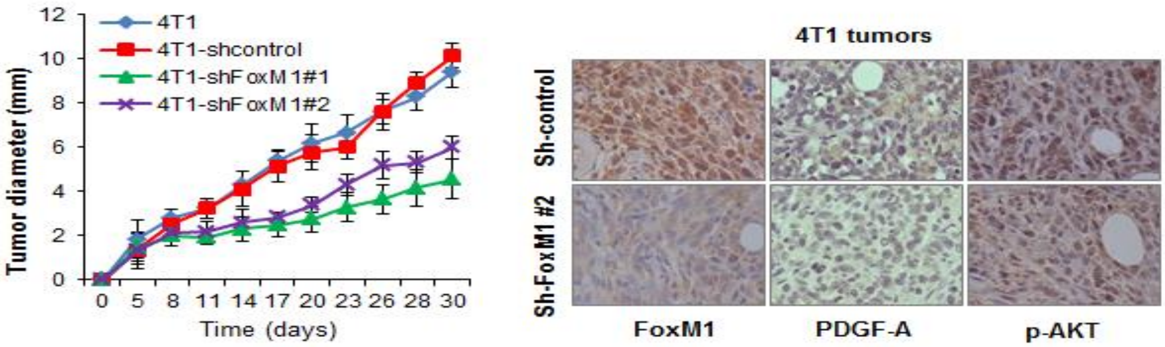

Supplementary Figure 6: 4T1 stable cells (5×10^3 per mouse) with or without shRNA-mediated depletion of FoxM1 were injected into the mammary fat pad of nude mice (n = 8 for each group). The tumor diameter was determined at different time points (left panel). The expression of FoxM1, PDGF-A, and p-AKT was analyzed by immunohistochemical analysis (right panel).

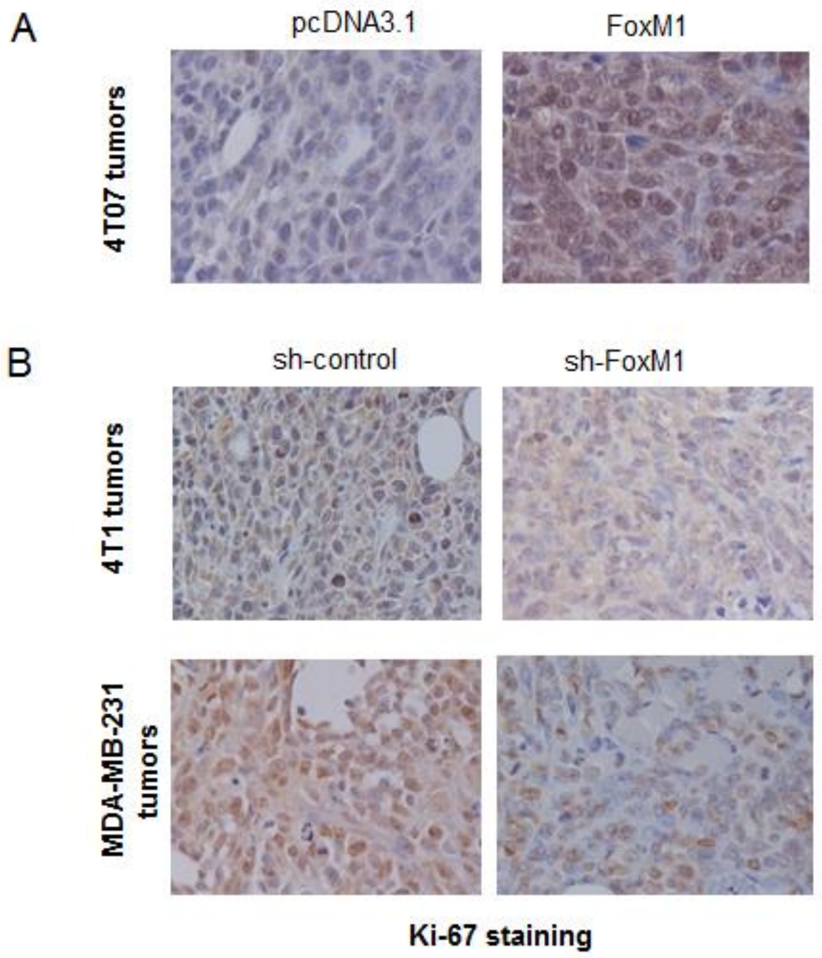

Supplementary Figure 7. IHC assays show the expression of Ki-67 in xenograft tumors of breast cancer cells. A, Tumors arose from 4T07-FoxM1 stable cells and the corresponding 4T07 control cells. B, Tumors arose from 4T1-sh-FoxM1 stable cells and MDA-MB-231-sh-FoxM1 stable cells. ×200 magnification.

Supplementary Table S1: Primers used for plasmid construction, electrophoresis mobility shift assay (EMSA), and chromatin immunoprecipitation (ChIP).

Primer Name	Sequence
FoxM1-RT-mouse	F: 5'-CACTTGGATTGAGGACCACTT-3'
	R: 5'-GTCGTTTCTGCTGTGATTCC-3'
PDGFA-RT-mouse	F: 5'-TCATTTACGAGATACCTCGG-3'
	R: 5'-TCCTGACATACTCCACTTTG-3'
GAPDH-RT-mouse	F: 5'-CTGGTATGACAATGAATA-3'
	R: 5'- CGAACTTTATTGATGGTA-3'
FoxM1-RT-human	F: 5'-AAGGTTGAGGAGCCTTCGAG-3'
	R: 5'-ATTCGGTCGTTTCT GCTGCTT-3'
PDGFA-RT-human	F: 5'- AAGTCCAGGTGAGGTTAG -3'
	R: 5'-TCCTCTTCCCGATAA T CC-3'
GAPDH-RT-human	F: 5'-CGGATTTGGTCGTATTGG-3'
	R: 5'-TCCTG GAAGATGGTGATG-3'
Mut-Site1 (−544 to −535 bp)	F: 5' –AGGTGAGTGCCCCGCACCCG-3'
	R: 5'-CGGGTGCGGGGCACTCACCT-3'
Mut-Site2 (−211 to −201 bp)	F: 5'- TTTGATGGATGCAGCTGCTTG-3'
	R: 5'-CAAGCAGCTGCATCCATCAAA-3'
EMSA-Site1	F: 5'-AGGTGAGTGCCCCAAACCCG-3'

	R: 5'-CGGGTTTGGGGCACTCACCT-5'
EMSA-Site2	F: 5'- TTTGATGGATTTAGCTGCTTG-3'
	R: 5'-CAAGCAGCTAAATCCATCAAA-3'
ChIP-Site1	F: 5'-CGACTGGAGCTCGCTCCC-3'
	R: 5'-GGGACCGGCTCTCTGGCC-3'
ChIP-Site2	F: 5'-GCCGCAGGATTGCAGCTG-3'
	R: 5'-CGAACCCCGAGCGCTTCC-3'

Supplementary materials

Quantitative real-time polymerase chain reaction (qRT-PCR)

Total RNA was isolated using TRIzol reagent (Invitrogen), according to the manufacturer's instructions. First strand cDNA was synthesized from total RNA (1 µg) using Moloney murine leukemia virus reverse transcriptase (Invitrogen). Relative expression of *FoxM1* or *PDGF-A* was determined by qRT PCR using iQ™

SYBR® Green Supermix (Bio-Rad). *GAPDH* was used for normalization. All experiments were repeated three times in triplicate. Primers used for reverse transcription and qRT PCR are shown in Supplementary Table S1.

Generation of stable cell lines

Stable transfection of breast cancer cells was performed as reported previously (16). To generate the FoxM1 stable expression cells, pcDNA3.1-FoxM1 was transfected into 4T07 and BT474 cells using FuGENE HD transfection reagent (Roche), and pcDNA3.1 was used as a control. To generate the FoxM1 or PDGF-A stable knockdown cells, pSilencer-shFoxM1 or pSilencer-shPDGF-A was transfected into MDA-MB-231 and 4T1 cells, and the pSilencer-shcontrol plasmid was used as a control. Stably transfected cell lines were isolated by neomycin (G418) selection.

Histologic, immunohistochemical, and immunofluorescence assays

Tissue sections of tumors were stained with hematoxylin and eosin according to standard protocols. For immunohistochemical analysis, tissue sections were incubated with antibodies against FoxM1 (sc-500, Santa Cruz Biotechnology), PDGF-A (NBP1-19781, Novus Biologicals), phospho-AKT (#9271, Cell Signaling Technology), or Ki-67 (sc-15402, Santa Cruz Biotechnology). For immunofluorescence analysis, tissue sections were first incubated with the primary antibodies of different origin and then incubated with secondary antibodies conjugated with Alexa Fluor 488 or Alexa Fluor 647 (Invitrogen). Images were taken under a fluorescence microscope (magnification ×200).
